# From open Ivor Lewis esophagectomy to a hybrid robotic-assisted thoracoscopic approach: a single-center experience over two decades

**DOI:** 10.1007/s00423-022-02497-6

**Published:** 2022-03-24

**Authors:** Fiorenzo V. Angehrn, Kerstin J. Neuschütz, Lana Fourie, Alexander Wilhelm, Silvio Däster, Christoph Ackermann, Markus von Flüe, Daniel C. Steinemann, Martin Bolli

**Affiliations:** Department of Visceral Surgery, Clarunis-University Center for Gastrointestinal and Liver Diseases, St. Clara Hospital and University Hospital Basel, Postfach, 4002 Basel, Switzerland

**Keywords:** Esophageal cancer, Ivor Lewis esophagectomy, Open esophagectomy, Robotic-assisted hybrid esophagectomy, Morbidity, Mortality

## Abstract

**Purpose:**

Robotic-assisted procedures are increasingly used in esophageal cancer surgery. We compared postoperative complications and early oncological outcomes following hybrid robotic-assisted thoracoscopic esophagectomy (Rob-E) and open Ivor Lewis esophagectomy (Open-E), performed in a single mid-volume center, in the context of evolving preoperative patient and tumor characteristics over two decades.

**Methods:**

We evaluated prospectively collected data from a single center from 1999 to 2020 including 321 patients that underwent Ivor Lewis esophagectomy, 76 underwent Rob-E, and 245 Open-E. To compare perioperative outcomes, a 1:1 case-matched analysis was performed. Endpoints included postoperative morbidity and 30-day mortality.

**Results:**

Preoperative characteristics revealed increased rates of adenocarcinomas and wider use of neoadjuvant treatment over time. A larger number of patients with higher ASA grades were operated with Rob-E. In case-matched cohorts, there were no differences in the overall morbidity (69.7% in Rob-E, 60.5% in Open-E, *p* value 0.307), highest Clavien-Dindo grade per patient (43.4% vs. 38.2% grade I or II, *p* value 0.321), comprehensive complication index (median 20.9 in both groups, *p* value 0.401), and 30-day mortality (2.6% in Rob-E, 3.9% in Open-E, *p* value 1.000). Similar median numbers of lymph nodes were harvested (24.5 in Rob-E, 23 in Open-E, *p* value 0.204), and comparable rates of R0-status (96.1% vs. 93.4%, *p* value 0.463) and distribution of postoperative UICC stages (overall *p* value 0.616) were observed.

**Conclusions:**

Our study demonstrates similar postoperative complications and early oncological outcomes after Rob-E and Open-E. However, the selection criteria for Rob-E appeared to be less restrictive than those of Open-E surgery.

**Supplementary Information:**

The online version contains supplementary material available at 10.1007/s00423-022-02497-6.

## Introduction

Esophageal cancer is the 8th most common cancer worldwide and the 6th leading cause of cancer death [1, 2].

Historically, squamous cell carcinoma was the predominant histological type worldwide. However, over the past decades, a demographic difference has emerged with squamous cell carcinomas being predominant in Asia and adenocarcinomas being more common in Europe and North America. This can be explained by the increased incidence of risk factors for adenocarcinoma such as gastroesophageal reflux disease and obesity in the western world [2, 3].

Overall, the majority of esophageal cancers are detected at a late stage, which severely affects treatment options [3]. Therapeutic regimens vary from local treatment for lesions that do not exceed the submucosa, to surgical esophagectomy as well as radio- and chemotherapy for more advanced tumor stages [2–4].

Within the last decades, the surgical approach has evolved, and new techniques including minimally invasive and robotic-assisted procedures have been adopted [5]. However, the literature is highly heterogenous with publications comparing different surgical techniques: open esophagectomies with laparotomy and thoracotomy, totally minimally invasive procedures with laparoscopy and thoracoscopy, and robotic-, laparoscopically, or thoracoscopically assisted esophagectomies, and include different approaches such as 2-stage Ivor Lewis and 3-stage McKeown procedures. Some of these studies show reduced rates of postoperative morbidity using totally minimally invasive or minimally invasive-assisted techniques while achieving the same oncological outcomes as open procedures [5–7].

In our center, Ivor Lewis esophagectomy has been routinely performed for the past 20 years [8, 9]. In 2015, we adopted a robotic approach for the thoracic phase of this operation. However, to obtain optimal gastric tube mobilization and preparation, we have continued to perform the abdominal phase of the Ivor Lewis esophagectomy using open surgery. This hybrid Ivor Lewis esophagectomy, open abdominal and a robotic thoracic phase, is currently the standard practice in our institution.

To provide a reliable comparison between these two surgical techniques, in this study, we analyzed postoperative morbidity and early oncological efficacy after hybrid robotic-assisted thoracoscopic Ivor Lewis esophagectomy (Rob-E) and open Ivor Lewis esophagectomy (Open-E), which were performed in a single mid-volume center, within the context of evolving preoperative patient and tumor characteristics over a period of 21 years.

## Methods

### Patients

The study included all patients that underwent Ivor Lewis esophagectomy for esophageal cancer from January 1999 to December 2020 at the University Center for Gastrointestinal and Liver Disease (St. Claraspital, Clarunis, Basel, Switzerland). Exclusion criteria were transhiatal or emergency procedures. All patients provided general consent, and the study was approved by the Ethical Committee of Northwestern Switzerland.

### Data collection

**Fig. 1:**
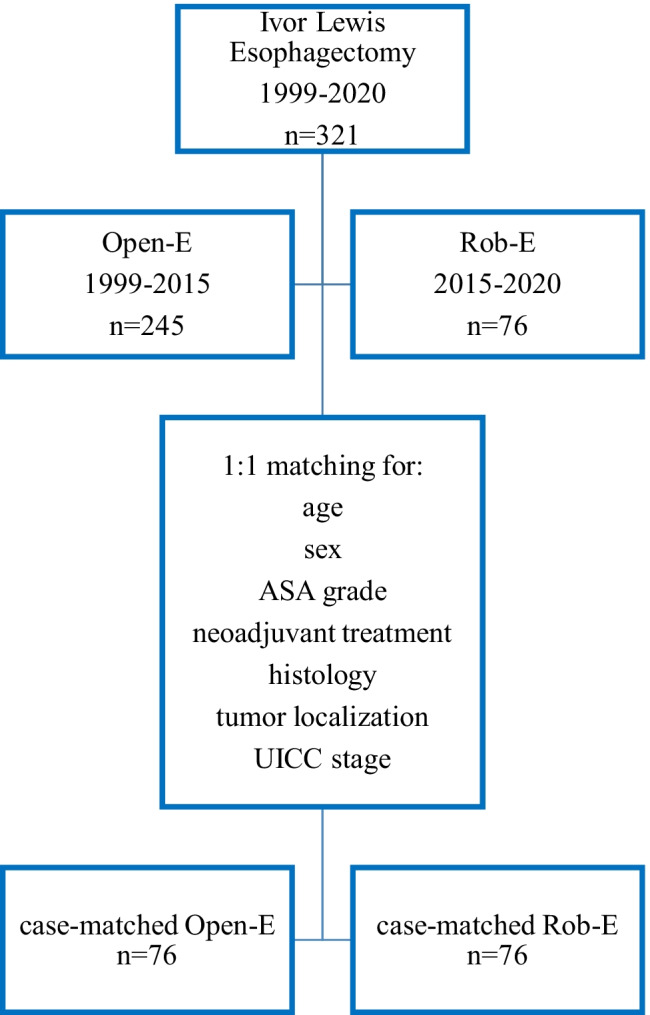
Flow chart of patients’ cohorts. A total of 321 patients were treated with Ivor Lewis esophagectomy between January 1999 and December 2020. From 1999 to 2015, an Open-E surgical procedure was performed in 245 cases. From 2015 to 2020, 76 patients underwent Rob-E surgery. To allow a reliable comparison of morbidities and oncological outcome, we selected a cohort Open-E patients which were 1:1 case-matched with Rob-E patients regarding the indicated preoperative characteristics. Postoperative morbidity and oncological outcomes were then evaluated in these case-matched cohorts

Data were prospectively collected and recorded in an institutional study registry database (Fig. 1). Raw data included patients’ characteristics such as age, sex, and American Society of Anesthesiologist (ASA) grade, pre- and postoperative tumor features including histological type, tumor localization and stage, (neo-)adjuvant therapy, postoperative morbidity, 30-day mortality, and duration of hospitalization.

### Study endpoints

Endpoints included the rate of postoperative complications as well as the 30-day mortality. Complications were defined as any event requiring a deviation of the therapeutic regimen. All complications occurring within the index hospitalization were recorded, and their severity was assessed using the Clavien-Dindo classification and the comprehensive complication index (CCI) [10, 11].

### Case-matched analysis

To compensate for significant differences in preoperative features observed between patients operated with Rob-E or Open-E, we performed a 1:1 case-matched analysis for age, sex, ASA grade, neoadjuvant treatment, UICC stage, histological tumor type, and tumor location (Table 2).

### Statistical analysis

Nominal and ordinal data are presented as counts and percentage, interval and ratio data are presented as median, lower and upper quartile for independent, non-normally distributed data. Analyses were performed using Fisher’s exact test and Mann–Whitney-*U* test as appropriate. For an adequate analysis of the postoperative details including the complications, a 1:1 case-matched analysis was performed regarding age, sex, ASA grade, neoadjuvant treatment, histology, tumor localization, and stage. *p* values < 0.05 were considered as statistically significant. Analyses were conducted using SigmaStat 4.0 and Stata/MP 17.

### Surgical technique

All surgeries were performed by two senior surgeons. In 2015, the first senior surgeon, who performed all Open-E surgeries until then transferred his skills to the second senior surgeon over a time of 1 year, in which both surgeons were present during all surgeries. The second senior surgeon was already very experienced and had performed numerous esophagostomies at another institution and over 500 robotic surgeries. Within another year, the transition to Rob-E was achieved by the second senior surgeon. Surgical steps in Rob-E were taught to fellow surgeons with a two-console concept to ensure teaching and education.

The Ivor Lewis esophagectomy consists of an abdominal phase for gastric mobilization, abdominal lymph node dissection, and construction of a tubular stomach and a thoracic phase for mediastinal lymph node dissection and the intrathoracic anastomosis.

**Fig. 2:**
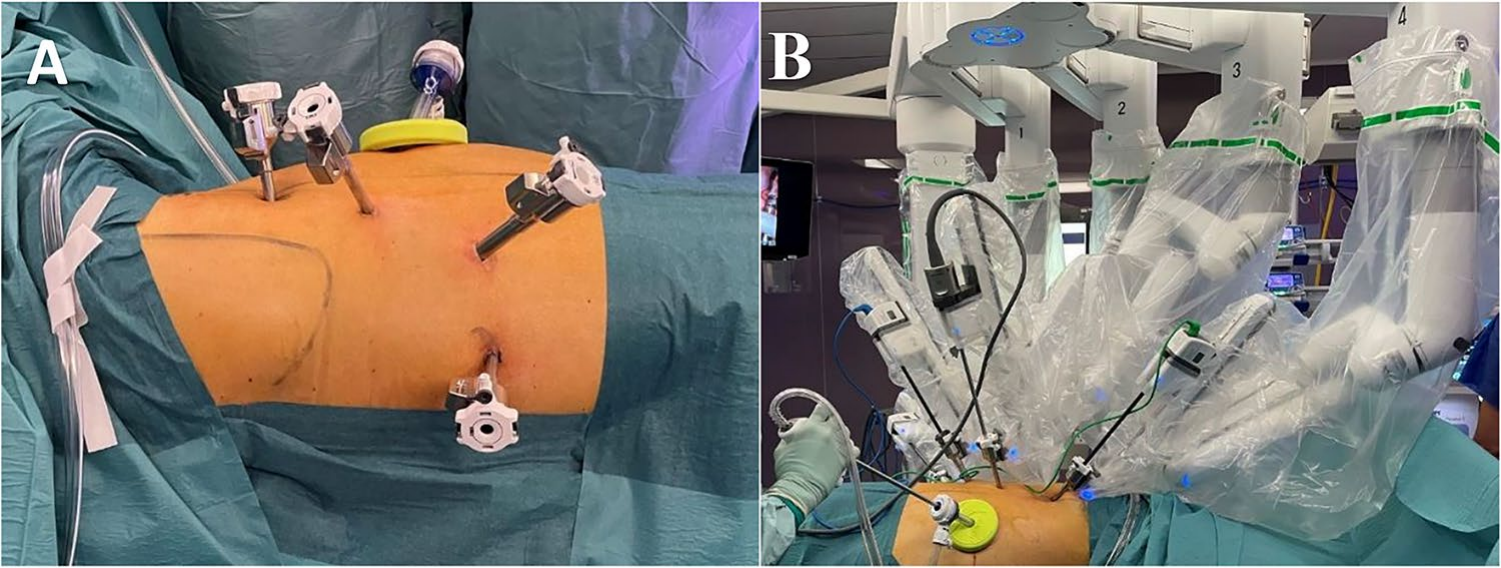
Port positioning and docking of the da Vinci Xi arms during the thoracic phase of Rob-E. **A** Dorsal view. Patient in left semi-prone position. Initially, a mini thoracotomy is preformed, a wound protector installed, and a 12-mm assistance port is inserted. The 1st robotic port is inserted in the posterior axillary line, being cautious not to interfere with the scapula. Additional robotic ports are inserted alongside a slope towards dorsal and caudal, maintaining a distance of at least 8 cm between the ports to avoid instrument collision. **B** Ventral view. Patient in left semi-prone position. The robotic arms are docked to the appropriate ports. A further instrument can be inserted through the assistance port

**Fig. 3:**
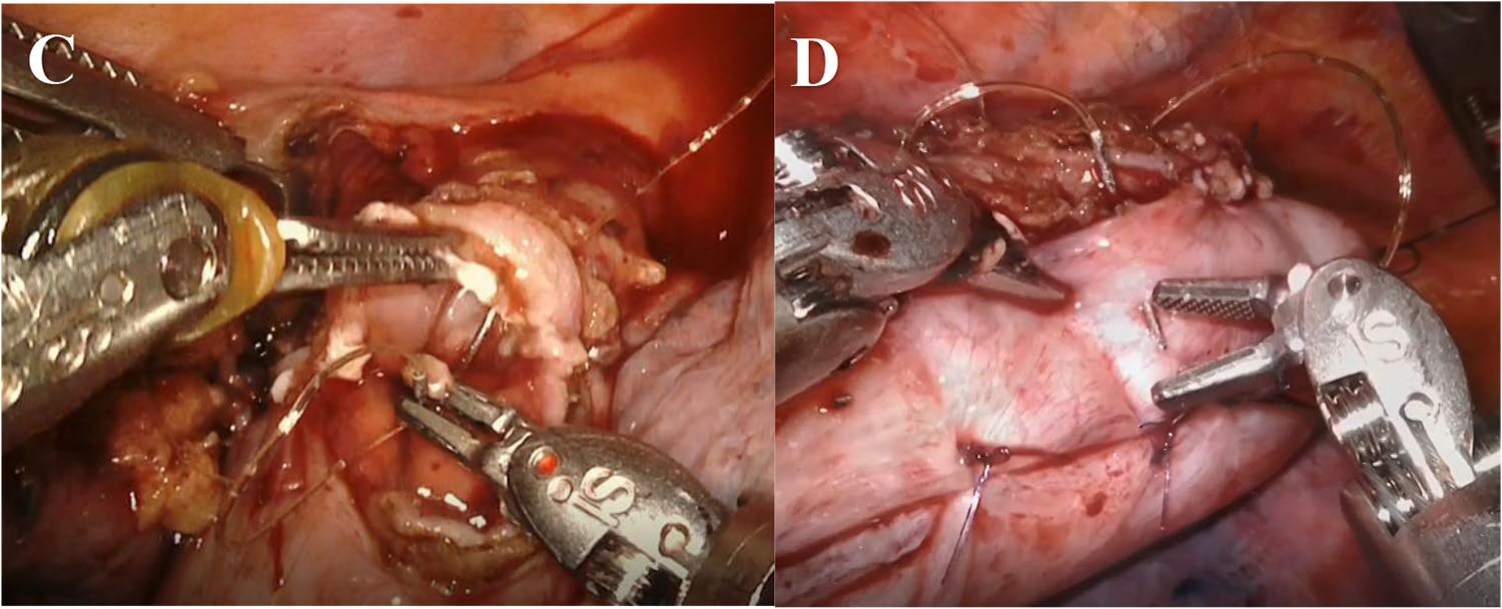
Continuously sutured anastomosis in Rob-E. **C**, **D** Continuously sutured anastomosis connecting the esophagus to the gastric conduit using a barbed suture. Completion of the posterior wall shown in **C** and of the anterior wall shown in **D**

In both Rob-E and Open-E, the abdominal phase was conducted via a median laparotomy. In Open-E, a right thoracotomy was performed during the thoracic phase [8, 9], whereas from October 2015, in Rob-E, this part was conducted using the da Vinci Xi robot (Fig. 2). During the thoracic phase of Rob-E, the right lung was excluded using a double-lumen endotracheal tube and isolated left lung ventilation. In Open-E, an end-to-end esophagogastrostomy was performed using a circular stapler. In Rob-E, the anastomosis was most frequently performed as an end-to-side esophagogastrostomy in a continuously sutured manor (Fig. 3). In Open-E, a pyloromyotomy was performed when the gastric tube was constructed; in Rob-E, this step was left out.

### Early oncological outcome evaluation

Postoperative oncological outcome was assessed by analyzing residual tumor status and the number of harvested lymph nodes. In addition, the postoperative UICC stage was also evaluated.

### Postoperative regimen

All patients were admitted to the intensive care unit postoperatively. Total parenteral nutrition was initiated, and a nasogastric tube was left in situ for 7 days postoperatively. Patients were transferred to the general surgical ward based on their clinical course. On the 7th postoperative day, a radiological control of the anastomosis was performed, followed by the removal of the nasogastric tube and administration of peroral liquids. Regular diet was resumed progressively.

## Results

**Fig. 4:**
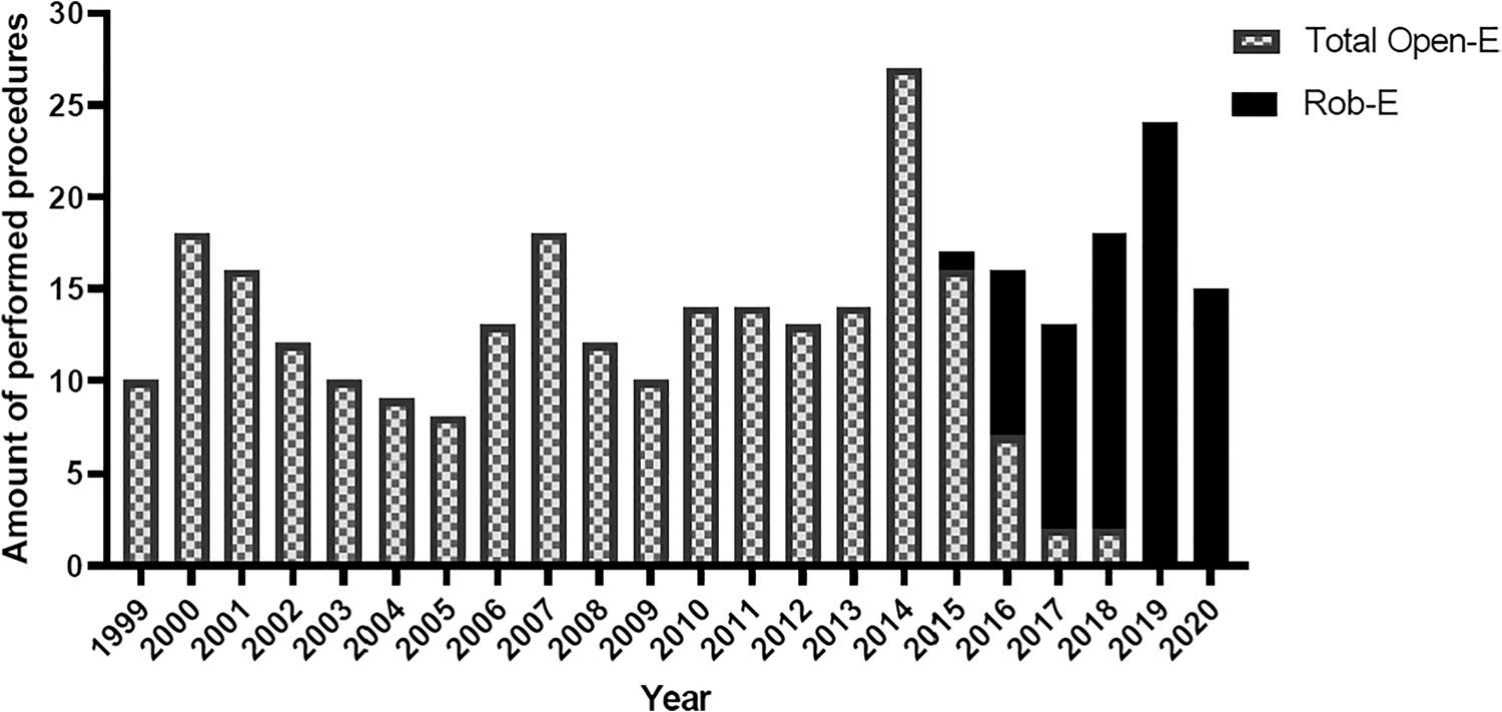
Annual amount of performed Ivor Lewis esophagogastrectomies. Amount of Ivor Lewis esophagogastrectomies in a mid-volume center. Introduction of Rob-E in 2015

Between January 1999 and December 2020, 321 patients underwent an Ivor Lewis esophagectomy for esophageal cancer at the University Center for Gastrointestinal and Liver Disease (St. Claraspital, Clarunis, Basel, Switzerland). A total of 245 patients were treated with an open procedure (Open-E), whereas 76 were treated with hybrid robotic-assisted surgery (Rob-E) (Fig. 1). Procedures per year and type of approach for unmatched surgeries are shown in Fig. 4. (More details on outcomes for the unmatched surgeries are provided in the supplemental material Table S1, Figure S1 and Figure S2.)

### Evolution of preoperative patient and tumor characteristics

Preoperative patient and tumor characteristics of all treated patients are shown in Table 1. There was a similar distribution of age and sex between Rob-E and Open-E with a median of 69.5 years vs. 67 years (*p* value 0.129) and 15.8% vs. 22.9% female patients (*p* value 0.203). However, ASA grades differed significantly (overall *p* value 0.007). In Rob-E, the most frequent ASA grade was III (64.5%), whereas in Open-E, most patients had an ASA grade of II (48.6%).

**Table 1:** Preoperative patient and tumor characteristics. Age is shown as median (lower and upper quartile), remaining data are shown as percentage (counts). UICC stage data are missing in 13.1% (*n* = 32) of Open-E

	Rob-E (***n*** = 76)	Open-E (***n*** = 245)	***p*** value
Age	69.5 years (63, 73.75)	67 years (59, 72)	0.129
Sex			
Male	84.2% (64)	77.1% (189)	0.203
Female	15.8% (12)	22.9% (56)	
ASA grade			
I	0.0% (0)	3.3% (8)	0.007
II	31.6% (24)	48.6% (119)	
III	64.5% (49)	41.2% (101)	
IV	3.9% (3)	6.9% (17)	
Histological type			
Adenocarcinoma	93.4% (71)	78.4% (192)	< 0.001
Squamous cell carcinoma	5.3% (4)	21.6% (53)	
Neuroendocrine tumor	1.3% (1)	0.0% (0)	
Tumor localization			
Upper esophagus	0.0% (0)	0.8% (2)	< 0.001
Middle esophagus	5.3% (4)	12.7% (31)	
Distal & Siewert I	59.2% (45)	73.1% (179)	
Siewert II	31.6% (24)	12.7% (31)	
Siewert III	3.9% (3)	0.8% (2)	
UICC			
I	19.7% (15)	21.6% (53)	0.893
II	26.3% (20)	16.7% (41)	
III	53.9% (41)	45.7% (112)	
IV	0.0% (0)	2.9% (7)	
Neoadjuvant treatment	82.9% (63)	55.9% (137)	< 0.001

As Rob-E was introduced in 2015 and Open-E was performed from 1999 to 2015, we were able to evaluate the evolution of preoperative characteristics over time. We observed a significantly higher rate of adenocarcinomas and lower rate of squamous cell carcinomas in Rob-E compared to Open-E (93.4% vs. 78.4% and 5.3% vs. 21.6%, overall *p* value < 0.001). Furthermore, neoadjuvant treatment rates were significantly higher in patients undergoing Rob-E compared to Open-E (82.9% vs. 55.9%, *p* value < 0.001).

In the Rob-E cohort, there were significantly more further aborally located tumors with a higher percentage of Siewert 2 [12] carcinomas (31.6% vs. 12.7%, overall *p* value < 0.001). Yet, the distribution of tumor stages according to the Union for International Cancer Control (UICC) classification was similar, with UICC III being most common in both cohorts (53.9% vs. 45.7%, overall *p* value 0.893).

### Case-matched analysis

The significant differences in preoperative features observed in our mid-volume center between patients operated with Rob-E or Open-E reflect the change in basic patient and tumor characteristics and esophageal cancer treatment regimen over the past two decades. However, this precluded a reliable comparative analysis of perioperative morbidity and short-term oncological outcome. The performed 1:1 case-matched analysis ensured an adequate comparison of these surgical techniques (Table 2).

**Table 2:** Preoperative patient and tumor characteristics in 1:1 case-matched cohorts. Age is shown as median (lower and upper quartile); remaining data shown as percentages (counts)

	Rob-E (***n*** = 76)	Open-E (***n*** = 76)	***p*** value
Age	69.5 years (63, 73.75)	70 years (60, 73)	0.900
Sex			
Male	84.2% (64)	84.2% (64)	1.000
Female	15.8% (12)	15.8% (12)	
ASA grade			
I	0.0% (0)	0.0% (0)	1.000
II	31.6% (24)	31.6% (24)	
III	64.5% (49)	64.5% (49)	
IV	3.9% (3)	3.9% (3)	
Histological type			
Adenocarcinoma	93.4% (71)	93.4% (71)	1.000
Squamous cell carcinoma	5.3% (4)	6.6% (5)	
Neuroendocrine tumor	1.3% (1)	0.0% (0)	
Tumor localization			
Upper esophagus	0.0% (0)	0.0% (0)	0.851
Middle esophagus	5.3% (4)	5.3% (4)	
Distal & Siewert I	59.2% (45)	61.8% (47)	
Siewert II	31.6% (24)	30.3% (23)	
Siewert III	3.9% (3)	2.6% (2)	
UICC			
I	19.7% (15)	19.7% (15)	0.707
II	26.3% (20)	22.4% (17)	
III	53.9% (41)	57.9% (44)	
IV	0.0% (0)	0.0% (0)	
Neoadjuvant treatment	82.9% (63)	81.6% (62)	1.000

A detailed list of postoperative complications as observed in the case-matched cohorts is shown in Table 3 (for all cases, see supplemental material Table S1). There were no significant differences in the overall morbidity with a percentage of 69.7% in Rob-E and 60.5% in Open-E (*p* value 0.307). Furthermore, there was no significant difference in the distribution of the highest Clavien-Dindo grade per patient with grade I or II being most common in both cohorts (43.4% vs. 38.2%, overall *p* value 0.321), or the comprehensive complication index with a median of 20.9 in both groups (*p* value 0.401). A 30-day mortality did not differ either (2.6% vs. 3.9%, *p* value 1.000).

**Table 3:** Postoperative complications in the 1:1 case-matched cohorts. Comprehensive complication index and duration of hospitalization stay are shown as median (lower and upper quartile). Remaining data are shown as percentages (counts)

	Rob-E (***n*** = 76)	Open-E (***n*** = 76)	***p*** value
Overall morbidity	69.7% (53)	60.5% (46)	0.307
Clavien-Dindo			
I and II	43.4% (33)	38.2% (29	0.321
IIIA	14.5% (11)	7.9% (6)	
IIIB	3.9% (3)	2.6% (2)	
IV	5.3% (4)	7.9% (6)	
30-day mortality	2.6% (2)	3.9% (3)	1.000
Comprehensive complication index	20.9 (0, 33.5)	20.9 (0, 29.6)	0.401
Surgical reintervention	5.3% (4)	7.9% (6)	0.745
Duration of hospitalization	20 days (17, 23.75)	18.5 days (16, 23)	0.368
Clavien-Dindo I and II			
Pneumonia	30.3% (23)	25.0% (19)	0.587
Pulmonary embolism	5.3% (4)	3.9% (3)	1.000
Arrhythmia	19.7% (15)	19.7% (15)	1.000
Urinary tract infection	5.3% (4)	7.9% (6)	0.745
Urinary retention	5.3% (4)	3.9% (3)	1.000
Oral candidiasis	6.6% (5)	2.9% (2)	0.442
Central line-associated infection	1.3% (1)	2.6% (2)	1.000
Central line-associated thrombosis	2.6% (2)	1.3% (1)	1.000
Infection of unknown origin	0.0% (0)	1.3% (1)	1.000
Wound infection	1.3% (1)	0.0% (0)	1.000
Wound dehiscence	0.0% (0)	1.3% (1)	1.000
Delirium	10.5% (8)	7.9% (6)	0.780
Colitis	1.3% (1)	1.3% (1)	1.000
Splenic infarction	3.9% (3)	1.3% (1)	0.620
Drug-induced rash	1.3% (1)	1.3% (1)	1.000
Pressure ulcer	0.0% (0)	1.3% (1)	1.000
Parotitis	0.0% (0)	1.3% (1)	1.000
Focal seizure	0.0% (0)	1.3% (1)	1.000
Neurapraxia	3.9% (3)	1.3% (1)	0.620
Clavien-Dindo III and IV			
Anastomotic insufficiency	7.9% (6)	5.3% (4)	0.745
Mediastinitis	3.9% (3)	1.3% (1)	0.620
Esophagobronchial fistula	2.6% (2)	0.0% (0)	0.497
Chylothorax	1.3% (1)	1.3% (1)	1.000
Pleural effusion	7.9% (6)	6.6% (5)	1.000
Pneumothorax	6.6% (5)	5.3% (4)	1.000
Severe pneumonia	3.9% (3)	5.3% (4)	1.000
Pleural empyema	5.3% (4)	1.3% (1)	0.367
Acute respiratory distress syndrome	1.3% (1)	0.0% (0)	1.000
Severe arrhythmia	1.3% (1)	3.9% (3)	0.620
Sepsis	2.6% (2)	5.3% (4)	0.681
Perisplenic fluid collection	1.3% (1)	0.0% (0)	1.000
Stroke	0.0% (0)	2.6% (2)	0.497
Wound infection	0.0% (0)	3.9% (3)	0.245

The rate of surgical reintervention was similar (5.3% vs. 7.9%, *p* value 0.745). In Rob-E, surgical reintervention was necessary in four cases of a pleural empyema, two of which were accompanied by an esophagobronchial fistula. In Open-E, surgical reintervention was necessary in six cases. In four cases due to an anastomotic leakage, one of which was accompanied by a pleural empyema, and another by a cutaneous wound infection. Furthermore, one case of a chylothorax accompanied by a cutaneous wound infection, and one further case of a wound infection also required surgical reintervention.

Regarding the rate of anastomotic insufficiencies, there were no significant differences (7.9% vs. 5.3%, *p* value 0.745). However, in Rob-E, all insufficiencies were treated with endoscopic insertion of a stent or an endo-sponge®, whereas in Open-E, all insufficiencies required surgical reintervention. For the latter, it is noteworthy that in one case in 2008, endo-sponge® therapy was not available at our institution; one case treated in 2018 showed failure to treatment with endo-sponge® and stent, and the other two cases were aggravated by the presence of a sepsis and a mediastinitis. There were no significant differences in the rates of esophagobronchial fistulas (2.6% vs. 0.0%, *p* value 0.487), chylothoraces (1.3% vs. 1.3%, *p* value 1.000), or pleural empyemas (5.3% vs. 1.3%, *p* value 0.367).

The most frequent complication in both cohorts was the Clavien-Dindo grade II pneumonia requiring antibiotic treatment (30.3% vs. 25.0%, *p* value 0.587). The duration of hospitalization was similar with a median of 20 and 18.5 days, respectively (*p* value 0.368). An intraoperative conversion from Rob-E to Open-E was never necessary.

### Early oncological outcomes

A comparison of postoperative oncological details revealed no significant differences in patients treated with Rob-E or Open-E, as shown in Table 4 (for all cases, see supplemental material Figure S2). The median number of harvested lymph nodes (24.5 vs. 23, *p* value 0.204), as well as the residual tumor status with a R0-status of 96.1% in Rob-E and 93.4% in Open-E (*p* value 0.463) were similar. The distribution of postoperative UICC stages was comparable with stages III and I being most common in both cohorts (30.3% vs. 31.6% and 30.3% vs. 30.3%, overall *p* value 0.616). Surgery was followed by an adjuvant therapy in 22.4% of Rob-E and 28.9% of Open-E (*p* value 0.458).

**Table 4:** Postoperative oncological details. Harvested lymph nodes shown as median (lower and upper quartile); remaining data shown as percentage (counts)

	Rob-E (***n*** = 76)	Open-E (***n*** = 76)	***p*** value
Harvested lymph nodes	24.5 (18.5, 32)	23 (19, 28)	0.204
UICC			
0	18.4% (14)	14.5% (11)	0.616
I	30.3% (23)	30.3% (23)	
II	19.7% (15)	22.4% (17)	
III	30.3% (23)	31.6% (24)	
IV	1.3% (1)	1.3% (1)	
Residual tumor			
R0	96.1% (73)	93.4% (71)	0.463
R1	3.9% (3)	5.3% (4)	
R2	0.0% (0)	1.3% (1)	
Adjuvant therapy	22.4% (17)	28.9% (22)	0.458

## Discussion

Esophageal cancer is a challenging disease, amongst others because of late diagnosis due to the lack of symptoms in early stages and demanding surgical treatment with several possible surgical approaches [13, 14]. Our data, collected over a period of more than 20 years, allowed us to investigate, from a single mid-volume center point of view, the evolution of the basic patient and tumor characteristics over time.

We observed an increased rate of adenocarcinomas at the expense of squamous cell carcinomas. Overall, squamous cell carcinoma is still the predominant histological type worldwide; however, current literature suggests a drift towards an increased incidence of adenocarcinomas in North America and Europe, which is consistent with our data [13–15]. Furthermore, in our center, there has been an increased rate of neoadjuvant treatments performed over time. This reflects the change in esophageal cancer treatment over the past decade, which has largely evolved towards a multimodal approach. Several studies have shown that the survival of resectable esophageal cancer can be improved by neoadjuvant therapy, which is therefore now emerging as the standard preoperative treatment [14, 16, 17].

Over the last two decades, surgical treatment of esophageal cancer has evolved significantly. A vast range of surgical approaches, including open procedures, minimally invasive techniques, or combinations of both is currently under evaluation. In the literature, advantages of minimally invasive techniques, including hybrid approaches, have been demonstrated [4–7], but general recommendations have not emerged yet, and the choice remains within the surgical team. Considering the high heterogeneity of published data, the evaluation of more homogeneous results from a single mid-volume institution might provide important insights.

Over a period of 21 years, we performed 16 procedures on average and consider ourselves a mid-volume institution (Fig. 4). All surgeries were performed by only two surgeons and make this study of special interest. Therefore, the presented dates are representative for a mid- to high-volume center as regards of surgery per surgeon and year. Good outcomes in comparison with the literature [18, 19] might be ascribed to the experience of a highly specialized team that developed over two decades.

In the past 5 years, we have adopted a standardized, hybrid, robotic-assisted approach for Ivor Lewis esophagectomy. Given the fact, that in our study the abdominal phase is conducted in the same manner regardless of surgical approach, the outcomes basically reflect the thoracic phase of the Rob-E. Our final goal for the future will be a total robotic resection and reconstruction which is in line with the German AMWF guidelines suggesting a total minimal invasive approach or a combination of minimally invasive and open techniques (hybrid techniques) [20]. We are convinced to have a better understanding of the single changes, which influence outcomes, when adoptions are made gradually, as compared to a direct change from Open-E to a totally minimally invasive technique. We decided to omit thoracotomy first, as it seems more invasive than laparotomy. In the future, with a minimally invasive technique for the abdominal part in experienced hands, we expect a further decrease in the rate of pulmonary complications.

Overall, our results may suggest that the introduction of the hybrid Rob-E technique in our center has made surgeons more confident in performing higher risk surgeries as larger numbers of patients with higher ASA grades were operated. Thus, the criteria that were applied for the patients’ eligibility for surgery appear to be less restrictive in Rob-E compared to Open-E, benefiting patients with advanced stages or increased comorbidities.

To compare the outcomes between Rob-E and Open-E more reliably, we generated case-matched cohorts, taking into account a number of important variables potentially impacting on postoperative morbidities and clinical outcome.

We could show that there are no significant differences related to the use of Rob-E or Open-E, with regard to overall complications and 30-day mortality**.** This is consistent with previously published studies comparing similar surgical techniques [21]. Moreover, our rates of complications such as anastomotic leaks, surgical reinterventions, and mortality for both Rob-E and Open-E are comparable to data from benchmarking reports [22, 23]. For this study, the definition of anastomotic leakage is an endoscopically proven leakage. Endoscopy was only performed in case of a suspicious upper GI-contrast study 7 days postoperatively or if leakage was clinically suspected within 30 days. A recent register study reports much higher leakage rates with 33% for hand-sewn anastomoses [18]. A direct data comparison is questionable because of a missing definition of leakage, severity of the leakage, therapeutic consequences, and time of leakage in this registry study. We presume that while the registry study overestimates leakage rates, we might not document all clinically silent leakages because of a missing routine postoperative endoscopy. The German Cancer Society (DKG) demands for a leakage rate below 15%. If the rate is higher, it is considered noticeable and further explanations are demanded [24]. This supports the presumption of overestimation in the register study.

Literature for thoracoscopic esophagectomy shows a trend towards circular stapled or semi-mechanical anastomosis instead of hand-sewn anastomosis [25–27]. Semi-mechanical anastomosis enlarges the cross-sectional area and might prevent postoperative strictures of the anastomosis. Some authors also promote semi-mechanical anastomosis for robotic resection [28], although this question has not yet been definitely clarified. However, there are groups including ourselves promoting a return to hand-sewn anastomoses in view of the robotic advantages such as enhanced range of motion [29]. It is presumed that stenosis is a late sequela of apparent or inapparent anastomotic leakage. Leakage rate of 7.9% after robotic-assisted esophagectomy in the current study is far below the rate of both the hand-sewn and the stapled group in the UGIRA registry [18]. However, this direct comparison must be interpreted very cautiously, since the anastomotic leak was not consistently defined in the same way. Furthermore, this study is missing long-term results, and therefore no conclusion can be drawn as to the incidence of stenosis.

Interestingly, while neoadjuvant treatment has widely been shown to be associated with better overall survival [14, 16], its effect on perioperative morbidity is described controversially in the literature [30, 31]. In this context, it is reassuring that similarly good results were obtained in both Rob-E and Open-E.

Limitations of our study should be acknowledged. These include the lack of long-term follow-up and randomization. Furthermore, the data were collected in a single mid-volume institution and therefore only represent a relatively small cohort. Furthermore, diagnostic methods and management of postoperative morbidity have also evolved over time. However, given that our data is from a single center over a long period of time, it reliably reflects the evolution of patient characteristics and adoption of minimally invasive esophageal cancer surgery.

## Conclusion

Our results show that a higher percentage of patients with ASA grade III was treated after the introduction of the hybrid robotic-assisted thoracoscopic procedure in our institution, demonstrating a lower threshold in determining patients’ eligibility for surgery. There were no significant differences in postoperative complications and early oncological outcomes when comparing Rob-E and Open-E. Hence, we consider both procedures safe and effective.

## Supplementary Information

Below is the link to the electronic supplementary material.

Supplementary file1 (DOCX 266 KB)

Supplementary file2 (DOCX 335 KB)

Supplementary file3 (DOCX 18 KB)
