# Supplementary material for: From open Ivor Lewis esophagectomy to a hybrid robotic-assisted thoracoscopic approach: a single-center experience over two decades
Source: Langenbecks Arch Surg. 2022 Mar 24;407(4):1421–30. doi: 10.1007/s00423-022-02497-6 (PMC9283174; doi:10.1007/s00423-022-02497-6)

**Figure S1.** Course of annual complications including overall morbidity (A), 30-day mortality (A), rate of surgical reintervention (A) and anastomotic leakage (A), comprehensive complication index (B) and duration of hospitalization (C). B and C are shown as box whisker plots with median, upper and lower quartile and minimum and maximum values. Data shown for all performed Ivor Lewis esophagogastrectomies (Open-E and Rob-E, unmatched cohorts) from 2006 to 2020.


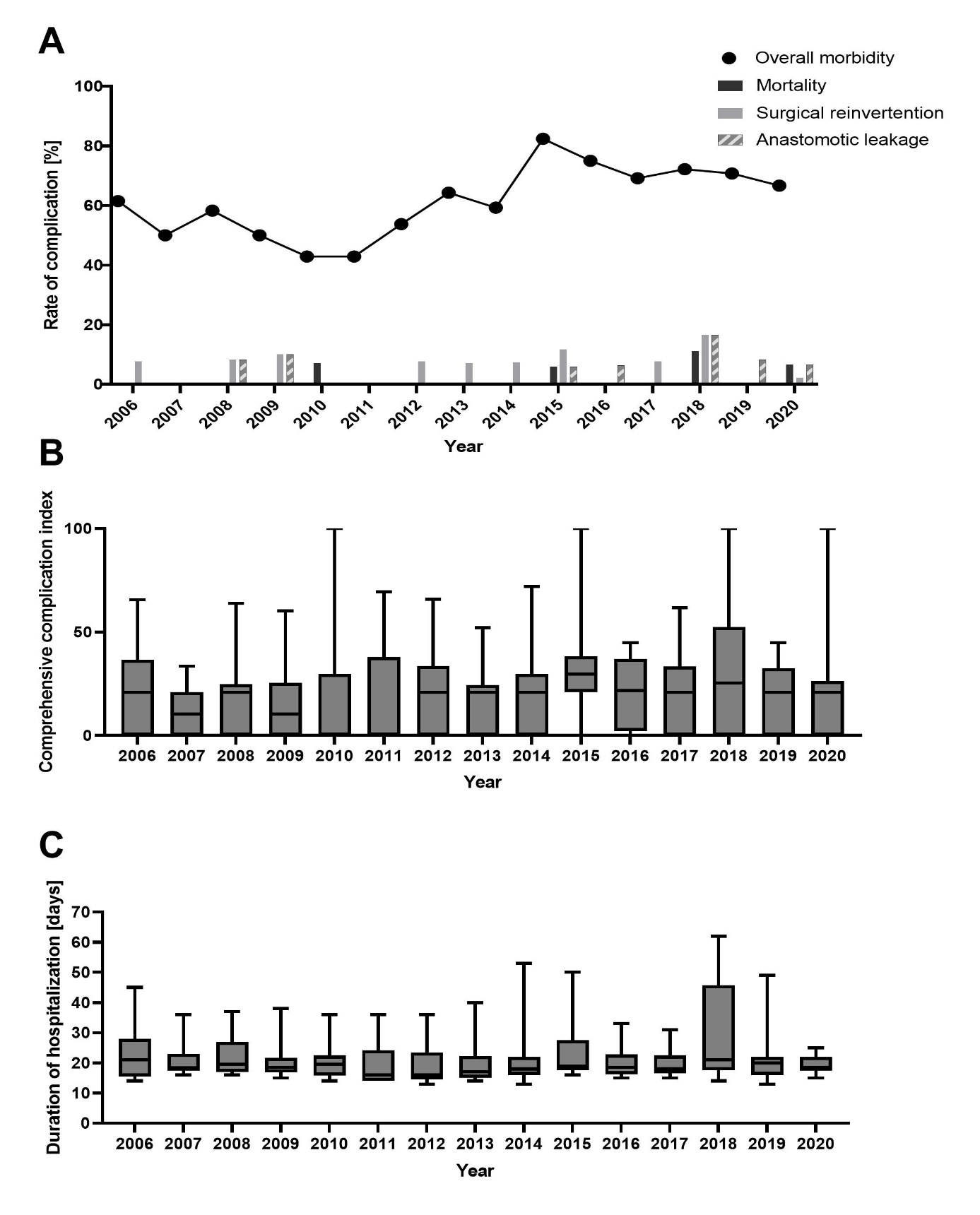

Supplement: Supplementary file 1 — Supplementary file1 (DOCX 266 KB) [file 423_2022_2497_MOESM1_ESM.docx]
