# Supplementary material for: From open Ivor Lewis esophagectomy to a hybrid robotic-assisted thoracoscopic approach: a single-center experience over two decades
Source: Langenbecks Arch Surg. 2022 Mar 24;407(4):1421–30. doi: 10.1007/s00423-022-02497-6 (PMC9283174; doi:10.1007/s00423-022-02497-6)
Supplement: Supplementary file 2 — Supplementary file2 (DOCX 335 KB) [file 423_2022_2497_MOESM2_ESM.docx]

**Figure S2.** Course of annual postoperative details including number of harvested lymph nodes (A), distribution of UICC stages (B) and R-status (C) as well as rate of adjuvant therapy (D). Figure A shown as box whisker plots with median, upper and lower quartile and minimum and maximum values. Data shown for all performed Ivor Lewis esophagogastrectomies (Open-E and Rob-E, unmatched cohorts) from 1999 to 2020.

**
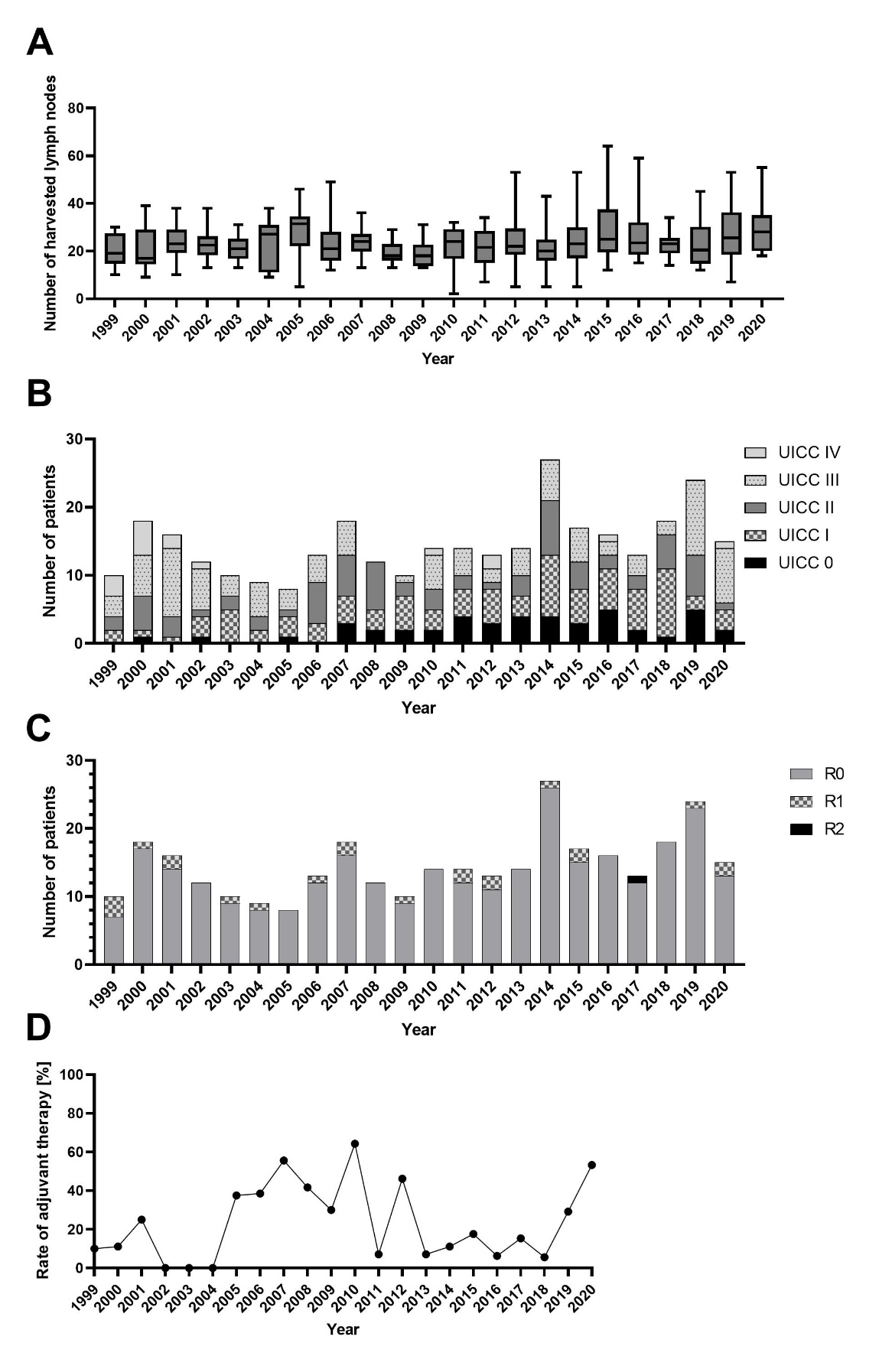
**
