# Supplementary material for: From open Ivor Lewis esophagectomy to a hybrid robotic-assisted thoracoscopic approach: a single-center experience over two decades
Source: Langenbecks Arch Surg. 2022 Mar 24;407(4):1421–30. doi: 10.1007/s00423-022-02497-6 (PMC9283174; doi:10.1007/s00423-022-02497-6)
Supplement: Supplementary file 3 — Supplementary file3 (DOCX 18 KB) [file 423_2022_2497_MOESM3_ESM.docx]

**Table S1:** Postoperative complications in the Rob-E, the case-matched Open-E and the total Open-E cohort for patients from 2006 to 2020. Comprehensive complication index and duration of hospitalization stay are shown as median (lower and upper quartile). Remaining data are shown as percentages (counts). Missing data in 83 (33.1%) patients of the total Open-E cohort (n=245) and therefore not included in this table. Hence, Open-E total_2006-2020_(n=245-83=162).

|  | | **Rob-E (n=76)** | **Open-E case-matched (n=76)** | **Open-E total**_2006-2020_ **(n=162)** |
| --- | --- | --- | --- | --- |
| Overall morbidity | | 69.7% (53) | 60.5% (46) | 58.6% (95) |
| Clavien-Dindo | |  |  |  |
| I & II | 43.4% (33) | 38.2% (29) | 34.6% (56) |  |
| IIIA | 14.5% (11) | 7.9% (6) | 13% (21) |  |
| IIIB | 3.9% (3) | 2.6% (2) | 2.5% (4) |  |
| IV | 5.3% (4) | 7.9% (6) | 6.8% (11) |  |
| 30-day mortality | | 2.6% (2) | 3.9% (3) | 1.9% (3) |
| Comprehensive complication index | | 20.9 (0, 33.5) | 20.9 (0, 29.6) | 20.9 (0, 29.6) |
| Surgical reintervention | | 5.3% (4) | 7.9% (6) | 6.2% (10) |
| Duration of hospitalization | | 20 days (17, 23.75) | 18.5 days (16, 23) | 18 (16, 23) |
| Clavien-Dindo I & II | |  |  |  |
| Pneumonia | 30.3% (23) | 25.0% (19) | 25.9% (42) |  |
| Pulmonary embolism | 5.3% (4) | 3.9% (3) | 4.3% (7) |  |
| Arrhythmia | 19.7% (15) | 19.7% (15) | 15.4% (25) |  |
| Urinary tract infection | 5.3% (4) | 7.9% (6) | 6.2% (10) |  |
| Urinary retention | 5.3% (4) | 3.9% (3) | 4.9% (8) |  |
| Oral candidiasis | 6.6% (5) | 2.9% (2) | 1.9% (3) |  |
| Central line associated infection | 1.3% (1) | 2.6% (2) | 2.5% (4) |  |
| Central line associated thrombosis | 2.6% (2) | 1.3% (1) | 0.6% (1) |  |
| Infection of unknown origin | 0.0% (0) | 1.3% (1) | 1.9% (3) |  |
| Wound infection | 1.3% (1) | 0.0% (0) | 1.9% (3) |  |
| Wound dehiscence | 0.0% (0) | 1.3% (1) | 0.6% (1) |  |
| Delirium | 10.5% (8) | 7.9% (6) | 6.2% (10) |  |
| Colitis | 1.3% (1) | 1.3% (1) | 0.6% (1) |  |
| Splenic infarction | 3.9% (3) | 1.3% (1) | 1.2% (2) |  |
| Drug-induced rash | 1.3% (1) | 1.3% (1) | 1.2% (2) |  |
| Pressure ulcer | 0.0% (0) | 1.3% (1) | 0.6% (1) |  |
| Parotitis | 0.0% (0) | 1.3% (1) | 0.6% (1) |  |
| Focal seizure | 0.0% (0) | 1.3% (1) | 0.6% (1) |  |
| Neurapraxia | 3.9% (3) | 1.3% (1) | 0.6% (1) |  |
| Transfusion-dependent anemia | 0.0% (0) | 0.0% (0) | 0.6% (1) |  |
| Clavien-Dindo III & IV | |  |  |  |
| Anastomotic insufficiency | 7.9% (6) | 5.3% (4) | 2.5% (4) |  |
| Mediastinitis | 3.9% (3) | 1.3% (1) | 0.6% (1) |  |
| Esophagobronchial fistula | 2.6% (2) | 0.0% (0) | 0.0% (0) |  |
| Chylothorax | 1.3% (1) | 1.3% (1) | 1.2% (2) |  |
| Pleural effusion | 7.9% (6) | 6.6% (5) | 12.3% (20) |  |
| Pneumothorax | 6.6% (5) | 5.3% (4) | 3.1% (5) |  |
| Severe pneumonia | 3.9% (3) | 5.3% (4) | 3.7% (6) |  |
| Pleural empyema | 5.3% (4) | 1.3% (1) | 1.2% (2) |  |
| Acute respiratory distress syndrome | 1.3% (1) | 0.0% (0) | 0.0% (0) |  |
| Severe arrhythmia | 1.3% (1) | 3.9% (3) | 2.5% (4) |  |
| Sepsis | 2.6% (2) | 5.3% (4) | 3.7% (6) |  |
| Perisplenic fluid collection | 1.3% (1) | 0.0% (0) | 0.0% (0) |  |
| Stroke | 0.0% (0) | 2.6% (2) | 1.9% (3) |  |
| Wound infection | 0.0% (0) | 3.9% (3) | 3.7% (6) |  |
